# Age Stratification in Acute Ischemic Stroke Patients with Heart Failure

**DOI:** 10.3390/jcm12010038

**Published:** 2022-12-21

**Authors:** Camron Edrissi, Chase Rathfoot, Krista Knisely, Carolyn Breauna Sanders, Richard Goodwin, Samuel I. Nathaniel, Thomas Nathaniel

**Affiliations:** 1 School of Medicine-Greenville, University of South Carolina, Greenville, SC 29605, USA; 2 Department of Biology, North Greenville University, Tigerville, SC 29688, USA

**Keywords:** stroke, ischemic, heart failure, age

## Abstract

**Background and Purpose**. Heart failure (HF) is considered one of the major risk factors associated with the severity of acute ischemic stroke(AIS). The risk factors associated with stroke severity in AIS with a history of HF is not fully understood. **Methods**. A prospectively maintained database from comprehensive stroke centers in PRISMA Health Upstate Sc, was analyzed for patients with AIS and a history of HF from January 2010 to 30 June 2016. The primary outcome was risk factors associated with a National Institute of Health Stroke Scale score (NIHSS) < 7 indicating lower severity and a score ≥ 7 indicating high severity for 65–74 age category and ≥75 years age category for AIS-HF patients. Univariate analysis was used to determine risk factors based on age categories and stroke severities, while multivariable analysis was used to adjust for the effect of confounding variables. **Results**: A total 367 AIS-HF patients were identified in this study, 113 patients were between 65–74 years old, while 254 patients were in the ≥75 years old age category. In the adjusted analysis for 65–74 age category, history of smoking (OR = 0.105, 95% Confidence interval (CI): 0.018–0.614, *p* = 0.012), triglycerides (Odd ratio(OR) = 0.993, 95% (CI): 0.987–0.999, *p* = 0.019), and International Normalized Ratio (INR) (OR = 0.454, 95% CI: 0.196–1.050, *p* = 0.045), and direct admission treatment (OR = 0.355, 95% CI: 0.137–0.920, *p* = 0.033) were associated with a lower stroke severity, elevated heart rate (OR = 1.032, 95% CI: 1.009–1.057, *p* = 0.007) was associated with a higher stroke severity. For the ≥75 years old age category, previous stroke (OR = 2.297, 95% CI: 1.171–9.852, *p* = 0.024), peripheral vascular disease (OR = 6.784, 95% CI: 1.242–37.065, *p* = 0.027), heart rate (OR = 1.035, 95% CI: 1.008–1.063, *p* = 0.012), and systolic blood pressure (OR = 1.023, 95% CI: 1.005–1.041, *p* = 0.012) were associated with a higher severe stroke severity. **Conclusions**: After adjusting for the effect of potential confounders, more risk factors were associated with a high severity of stroke among ≥75 years old compared with 65–74 years old AIS-HF patients. Elevated heart rate was an independent risk factor associated with stroke severity in 65–74 and ≥75 years old AIS-HF patients. Elevated heart rate and other identified risk factors should be managed to reduce stroke severity among elderly AIS-HF patients.

## 1. Introduction

Stroke is regarded as a disease of the elderly, considering that the incidence of stroke dramatically increases with age [1]. Furthermore, increased age is associated with poorer stroke outcomes [2]. Elderly patients are more likely to be discharged to a nursing institution rather than to return home and are more likely to be severely disabled three months following a stroke when compared to younger populations [2]. These findings are particularly pronounced in populations over 75 years of age, with a 2.5 times greater mortality rate than younger patients below age 75 [3].

More than 85% of all strokes occur in populations over 65 years old, while 72% occur in those ≥75 years [4]. Patients <65 years are reported to present with less severe stroke and better outcomes when compared to ≥75 years of age group [5,6]. This finding suggests that specific age-related risk factors may differentially contribute to stroke severity in patients 65 and 74 years old when compared with those ≥75 years.

Some stroke risk factors, including diabetes, hypertension, atrial fibrillation, coronary and peripheral artery disease, and heart failure, increase with age [3]. While the effect of the risk factors is not of the same magnitude in predicting the occurrence of stroke across all age groups, the severity of stroke conferred by the different risk factors often clusters among older adults, especially the ones with heart failure (HF) and significantly contribute to the severity of stroke [7].

Heart failure is a leading cause of morbidity, hospitalization, and mortality in older adults [8]. Most patients with HF are elderly, and HF is the most common diagnosis in elderly hospitalized patients [9,10,11]. HF is a risk factor for AIS, indicating that HF and stroke coexist and share common risk factors [12]. The risk of AIS is two to three times higher in patients with HF than in those without [13]. Therefore, stroke severity is expected to be higher in AIS-HF patients compared with AIS patients without HF. However, how HF and other risk factors contribute to stroke severity in older patients is not fully understood. This is because most studies regarding stroke in the elderly population did not differentiate between risk factors associated with 65–74 years old patients where the stroke is reported to be less severe compared with ≥75 where stroke severity is higher [5,6].

According to National Institutes of Health Stroke Scale (NIHSS) scores, stroke severity is categorized into mild (NIHSS score: ≤7), moderate (NIHSS score: 8–16), and severe (NIHSS score: ≥17) [14]. The probability of higher stroke severity is reported to be greater in patients with a score of >7 (more than 60.0%) than with a score of ≤7 (14.8%) [15]. A sharp difference and a lower severity was also observed at a threshold of ≤7 [16,17]. Therefore, understanding the risk factors contributing to stroke severity may help determine the characteristics of patients likely to show neurological changes during the first 48 h after the onset of AIS, especially in the older patient population.

Despite abundant research focused on risk factors for incidents of AIS and HF, the data on risk factors on stroke severity in AIS-HF 65–74 years old patients and compared with ≥75 is not fully understood. The prevalence of stroke is higher in populations older than 65 years, and there is a greater likelihood of poorer outcomes in populations 75 years and older. Therefore, risk factors associated with stroke severity in AIS-HF 65–74 years old may differ from those ≥75 years old. Because of the greater likelihood of poorer outcomes in populations ≥75 years, we hypothesize that more risk factors may contribute to worse neurologic functions in AIS-HF ≥ 75 years old compared with the 65–74 age group.

Our first goal is to identify risk factors associated with initial stroke severity in an elderly AIS population with HF stratified by 65–74 and ≥75 age categories. Our second goal is to determine whether these risk factors are different between ≥75 and 65–74 years old AIS-HF population. Findings from this study may provide further insight into the understanding of risk factors associated with stroke severity in AIS-HF patients and aid in clinical decision-making by identifying risk factors that can be managed to improve the care of AIS-HF patients.

## 2. Materials and Methods

### 2.1. Study Population

This observational study uses data of AIS-HF patients extracted from the Prisma health Stroke registry of patients treated between January 2010 and January 2016 at a regional stroke center in the United States. The inclusion criteria for the study are AIS-HF patients between 65–74 years old and those >75 years, fulfilling the definition of ischemic stroke (an episode of neurological dysfunction caused by focal cerebral, spinal or retinal infarction). Patients with hemorrhagic stroke, neurological deficits following trauma, infection, inflammation, and demyelination were excluded. Data collected for risk factors included atrial fibrillation, coronary artery disease, carotid artery stenosis, depression, diabetes, drug and alcohol use, dyslipidemia, stroke family history, heart failure, hypertension, migraines, obesity, previous stroke, previous transient ischemic attack (TIA), prosthetic heart valve, peripheral vascular disease, chronic renal disease, sleep apnea, and smoking. Medication history variables included blood pressure-reducing medications, cholesterol reducers, diabetic medications, and anti-depressants. Lab value variables were collected, including total cholesterol, triglycerides (TGL), high-density lipoprotein (HDL), low-density lipoprotein (LDL) lipids, blood glucose, serum creatinine, systolic blood pressure, and diastolic blood pressure. Stroke severity at admission was documented using the National Institute of Health Stroke score(NIHSS) and stratified as ≤7 to indicate a less severity while >7 indicates patients with higher stroke severities.

Data on ambulation status before, during, and after admission was extracted. Changes in ambulatory status were determined. Changes in ambulation at different times during the entire clinical history: on admission, during admission, and after discharge were determined. Scoring of ambulatory data was performed in this fashion: 0 (not documented); 1: (patients not able to ambulate); 2: (able to ambulate with assistance) 3: (able to ambulate independently). Changes in ambulation was quantified by taking their score at discharge and subtracting their ambulation score on admission, with greater than zero being an improvement in ambulation.

### 2.2. Statistical Analysis

For the univariate analysis, we used the student′s t-test (independent, two-tailed) for continuous variables, while chi-squared test was used for discrete variables, and differences in proportions were determined. The results for the continuous analysis were presented as mean and standard deviation, while categorical variables′ results were presented as percentages. The univariate analysis was also performed to further stratify each age group (i.e., 65–74 and ≥75) into AIS-HF patients with NIHSS ≤ 7 versus NIHSS > 7 during admission.

Binary logistic regression models were built using the established predictors from the univariate analysis. Variables were cross tabulated to discard multicollinearity. In addition, we repeated the test for non-categorized continuous variables and separately performed the analysis to detect possible statistical bias. Factors with a *p*-value < 0.3 were chosen to identify independent predictors of higher NIHSS scores that were approaching significance and, theoretically, more likely to significantly contribute to the model. Moreover, it helps to minimize discrepancies due to non-comparable parameters.

The binary logistic regression analysis was then performed by backward selection method using significant predicted variables from the univariate analysis. The back selection method was chosen because it allows us to include all the initially selected risk factors that were approaching significance in the model and then systematically removed if they did not contribute to the overall significance of the model.

For each binary regression model, the dependent variable was the NIHSS score stratification. The primary independent variables were risk factors, laboratory values, and ambulation status stratified by age 65–74 and ≥75 years old. Odds ratios and 95% confidence intervals (95% CIs) of outcome measures were obtained from this model with a significance of *p* < 0.05. The odds of AIS-HF patients presenting a higher stroke severity (NIH score > 7) were analyzed separately for the 65–74 years old and ≥75 years old. We then identified odds ratios of the identified independent variables that significantly predicted a lower or higher stroke severity. The sensitivity, specificity, and accuracy of the logistic regression model for both groups were analyzed using the correct classification percentage and area under the Receiver Operating Curve (ROC). The Hosmer-Lemeshow test and correlations determined multicollinearity and interactions among the independent variables. Statistical analyses were done using the Statistical Package for Social Sciences v 26.0 for Windows (SPSS, Chicago, IL, USA).

## 3. Results

Of the total 367 AIS-HF patients, 113 patients were between 65–74 years old, while 254 patients were ≥75 years old. Table 1 presents the risk factors of AIS patients stratified by age categories (65–74 years old vs. >75 years old). Table 1 presents risk factors of AIS-HF patients who are 65 to 74 versus ≥75 years old stratified by stroke severity. Among ≥75 years old AIS-HF patients, carotid artery stenosis, systolic and diastolic blood pressures, and rtPA were associated with a higher stroke severity (NIHSS > 7) on admission. In contrast, TGL and independent ambulation on admission or during discharge was associated with less stroke severity (NIHSS ≤ 7). For the 65–74 AIS-HF patients, peripheral vascular disease, systolic blood pressure, and rtPA were associated with higher stroke severity. In contrast, independent ambulation on admission and discharge was associated with lower stroke severities.

The result of the adjusted analysis for the AIS-HF population in the 65–74 and ≥75 age category is presented in Table 2. As shown in Table 2, peripheral vascular disease, heart rate, systolic blood pressure rate were associated with patients in the ≥75 age category, while history of smoking, serum creatinine and body mass index were associated with 65–74 age category. The predictive capability of the model was strong as shown by the AUC = 0.680, 0.633–0.727, *p* < 0.01).

Risk factors of AIS-HF population for the 65–74 years old age category is presented in Table 3. History of smoking, triglycerides, International Normalized Ratio (INR) and direct admission for treatment were associated with a lower stroke severity, while elevated heart rate was associated with a higher stroke severity.

The result of the adjusted analysis for the AIS-HF population in the ≥75 years old age category is presented in Table 4. Previous stroke, peripheral vascular disease, heart rate and systolic blood pressure were associated with a higher severe stroke severity.

## 4. Discussion

Age and HF are significant risk factors for AIS, and more than 70% of ischemic strokes occur in patients greater than 75 years old [18]. While many risk factors have been linked to AIS and HF, how the different risk factors contribute to stroke severities among AIS-HF patients in the elderly AIS-HF patients is not fully understood. In our adjusted analysis, we found that in the ≥75 years old AIS-HF patients, an elevated heart rate was associated with higher stroke severity. This finding was also observed in the adjusted analysis for the total AIS-HF population and the 65–74 age category of AIS-HF patients. Lower heart rates are associated with reduced hospitalizations and decreased mortality among HF patients [19,20]. Among older patients, optimal heart rate is reported to be between 70–76, and for every 10-point increase in heart rate, the risk of poor outcomes increases by 10% [19,21]. This increase is reported to be independent of β-blocker use [22] and associated with a decrease in cardiac perfusion to myocardium exacerbating dysfunction [23]. The explanation is that heart rate increases as a compensatory mechanism acutely during ischemic stroke, and this has been associated with poor neurologic outcomes and larger infarcts [24]. Moreover, lower heart rates are associated with better functional outcomes in stroke recovery [25]. In our current study, heart rate for 65–74 years old is 83.3–87.6 and 76.9–80.6 for ≥75 years AIS-HF patients and comparable to other studies in AIS patients with poor outcomes [24,26,27]. The link between elevated heart rate and the observed stroke severity in our AIS-HF patient population may be due to the compensatory rise in heart rate mechanisms and their inability to compensate. A similar explanation was provided for the poor neurologic outcomes observed in AIS with elevated heart rates [26,28].

We observed that in the 65–74 years old AIS-HF patients, a history of smoking, lower levels of TGLs, INR, and direct admission for treatment were associated with lower stroke severity. Our finding that lower INR was associated with a lower stroke severity has been reported by another study [29]. While the use of warfarin, the medication for controlling coagulation by itself, is not an absolute contraindication to recombinant tissue plasminogen activator(rtPA) administration; because of increased bleeding risk, an INR of >1.7 is a contraindication for the use of rtPA for AIS patient [30,31,32,33]. Nevertheless, stroke is reported to occur in patients on warfarin despite anticoagulation [34]. In addition, INR is also reported to be a good indicator of the likelihood of ischemia [35]. Therefore, optimizing *a* patient’s INR therapeutic range improves anticoagulation, prevents neurological deficits, and reduces stroke severity [36].

Although smoking is an apparent risk factor for AIS, some studies [31,37,38] have found an association between smokers that receive thrombolytics and improved clinical outcomes. The most common critique of studies that affirm the paradox is their small sample size [39], and most metanalyses find no association between smoking status and stroke severity [40,41]. The risk of stroke is reported to decrease after 2 to 4 years of smoking cessation and returns to the level of non-smoking status after five years of smoking cessation [25]. Smoking increases the risk of stroke in the short term by promoting thrombosis [42] and reducing cerebral blood flow via arterial vasoconstriction [43]. However, the thrombotic process can be reversible [44], and cerebral blood flow can significantly improve soon after quitting [25,39,45,46]. It is also possible that our AIS-HF population 65–74 with a previous smoking history showed a lower stroke severity returned to non-smoking status, and might have quit smoking for a significant period following the onset of AIS. Future studies on the effect of changes in smoking behavior (e.g., smoking cessation) and AIS-HF could help understand the relationship between smoking and stroke severity in 65–74 years old AIS-HF patients.

The few studies [47,48] that evaluated the association between serum TGLs and outcomes in AIS reported diverse associations, and lower total triglyceride levels were reported to be associated with a worse prognosis in AIS. While patients with end-stage heart failure are reported to present with low TGLs [49,50], a normal range of TGL is regarded to be too low for these patients [51,52]. Triglyceride levels have been shown to drop substantially during extreme stress as this is the metabolically active form of cholesterol [53]. Moreover, low levels of TGL lead to decreased ability to deal with oxidative stress and a loss of cell membrane integrity [54]. In our current study, higher total serum TGLs levels were associated with a reduced stroke severity among 65–74 years old AIS-HF patients even after adjustment for confounding variables. Our finding is supported by a previous study [55], where higher fasting TGL concentration was associated with better functional outcomes after stroke. The exact mechanisms behind the association of higher TGL and a reduced stroke severity in 65–74 years old AIS-HF patients need to be further explored, as this might be helpful for the treatment of AIS-HF patients.

Our finding that AIS-HF population 65–74 years old age category that were directed admitted for treatment were associated with a reduced stroke severity have been reported by other studies [56,57]. Direct admission is reported to decrease door-to-care and door-to-needle times to less than an hour in most AIS patients [58].Decreased times are associated with improved neurologic outcomes [59]. In elderly patients, direct admission leads to reduced mortality with direct admission and treatment within 90 min [57].

We observed that in the AIS-HF population of the ≥75 years old age category, previous stroke, peripheral vascular disease, heart rate, and systolic blood pressure were associated with severe stroke severity. A similar finding has previously been reported among older AIS patients by previous studies where a previous stroke history was reported to be an independent risk factor for poor prognosis of ischemic stroke [7,60]. In addition, the severity, mortality and disability rates of patients with a previous stroke history were significantly higher than that of first-ever stroke patients [60]. Moreover, poor neurologic outcomes has been reported in many patients with a previous of stroke [61,62,63,64]. This finding supports our current result of a higher stroke severity among AIS-HF patients in the ≥75 years category with a previous history of stroke. Furthermore, it emphasizes the importance of secondary prevention of ischemic stroke among AIS-HF patients.

We found that PVD was associated with higher stroke severity in the whole AIS population of 65–74 years and ≥75 years AIS-HF patients, and this was sustained in the AIS-HF patients in the ≥75 years category. PVD is a risk factor for all cardiovascular events, including stroke, and has been associated with increased mortality and poor outcomes in AIS patients [65]. While previous studies indicate that PVD is an independent predictor of poor outcomes in AIS patients [66,67], our current finding indicates that PVD is also associated with higher stroke severity in AIS-HF patients in the ≥75 years category. This finding suggests the role of PVD as an important predictor of stroke severity that needs to be sufficiently investigated in managing AIS-HF patients of all ages.

The association of systolic blood pressure (SB)P with a higher stroke severity was observed in our univariate analysis. Even after adjusting for confounding variables, the effect was sustained in the AIS-HF ≥ 75 years category. Elevated blood pressure (BP) is defined as systolic BP ≥ 140 mm Hg or diastolic BP ≥ 90 mm Hg [68], and high systolic BP is associated with unfavorable short-term functional status and long-term mortality in elderly patients [68]. In the current study, SBP values ranged between 147.98–156.56 mm Hg and were associated with higher stroke severity in the AIS-HF ≥ 75 years old category. It seems that elevated average SBP levels in our current study may reflect the stroke severity in AIS-HF ≥ 75 years population. Future studies on the effect of elevated SBP on short- and long-term higher stroke severity among elderly AIS-HF populations will be helpful in the development of management plans for the care of elderly AIS-HF populations.

Our study has several limitations. First, this is an observational study, which may generate biases. Second, this is a single-center study, which may limit the generalization of the results to other populations. Third, we did not have information on elevated SBP on short- and long-term stroke severities, and in our AIS-HF population, we analyzed data between 24–48 h of stroke onset. Fourth, data regarding long-term functional outcomes were unavailable; therefore, we could not analyze the effect of specific risk factors on long-term functional status and stroke severity. In addition, we do not have information about patients with a previous history of smoking that returned to being non-smokers and might have quit smoking. Therefore, we could not analyze the effect of changes in smoking behavior (e.g., smoking cessation) and AIS-HF to understand the relationship between smoking and stroke severity in elderly AIS-HF patients.

## 5. Conclusions

The present study revealed that in the AIS-HF population 65–74 years old, history of smoking, lower levels of triglycerides, INR, and direct admission for treatment were associated with a lower stroke severity, while heart rate was associated with a higher stroke severity. Whereas in the AIS-HF ≥ 75 years category, previous stroke, peripheral vascular disease, heart rate and systolic blood pressure were associated with a higher stroke severity.

## Figures and Tables

**Table 1:** Risk factors of acute Ischemic stroke patients with heart failure in patients 65 to 74 versus ≥75 years old are stratified by stroke severity. Comparison of Demographics and Clinical Characteristics of Acute Ischemic Stroke Patients with Heart Failure in patients who are 65 to 75 versus greater than 75 years old to evaluate for Stroke Severity. The continuous variables are represented as Mean ± SD, while discrete data are shown as percentages.

	65 to 74 Years Old			Greater Than ≥75 Years Old		
Characteristic	NIHSS ≤ 7	NIHSS > 7		NIHSS ≤ 7	NIHSS > 7	
Number of patients	61	52	*p*-value	105	149	*p*-value
Age Group: No. (%)						
<50 years	0	0	0.748	0	0	0.037 * ^a^
50–59	0	0		0	0	
60–69	30 (49.2)	24 (46.2)		0	0	
70–79	31 (50.8)	28 (53.8)		34 (32.4)	31 (20.8)	
>=80	0	0		71 (67.6)	118 (79.2)	
Age Mean ± SD	69.98 ± 3.16	70.02 ± 3.32	0.954	82.98 ± 4.96	84.58 ± 5.45	0.018 * ^b^
Race: No (%)						
White	45 (73.8)	41 (78.8)	0.790	91 (86.7)	127 (85.2)	0.342
Black	14 (23.0)	10 (19.2)		14 (13.3)	19 (12.8)	
Other	2 (3.3)	1 (1.9)		0	3 (2.0)	
BMI: Mean ± SD	30.89 ± 8.15	30.44 ± 9.41	0.787	26.85 ± 6.00	25.92 ± 6.01	0.232
Gender (%)						
Female	34 (55.7)	65 (61.9)	0.435	24 (46.2)	107 (71.8)	<0.001 * ^a^
Male	27 (44.3)	40 (38.1)		28 (53.8)	42 (28.2)	
Medical History: No. (%)						
Atrial Fib	26 (42.6)	22 (42.3)	0.973	57 (54.3)	93 (62.4)	0.194
Coronary Artery Disease	44 (72.1)	28 (53.8)	0.044 * ^a^	56 (53.3)	81 (54.4)	0.871
Carotid Artery Stenosis	5 (8.2)	5 (9.6)	0.791	5 (4.8)	18 (12.1)	0.045 * ^a^
Depression	11 (18.0)	11 (21.2)	0.676	19 (18.1)	24 (16.1)	0.677
Diabetes	32 (52.5)	26 (50.0)	0.794	41 (39.0)	64 (43.0)	0.534
Drugs or Alcohol	1 (1.6)	2 (3.8)	0.467	1 (1.0)	1 (0.7)	0.803
Dyslipidemia	42 (68.9)	35 (67.3)	0.861	57 (54.3)	87 (58.4)	0.516
Stroke Family History	7 (11.5)	2 (3.8)	0.135	7 (6.7)	3 (2.0)	0.060
Heart Failure	61 (100)	52 (100)		105 (100)	149 (100)	
Sleep apnea	1 (1.6)	4 (7.7)	0.119	5 (4.8)	5 (3.4)	0.570
Hypertension	53 (86.9)	48 (92.3)	0.351	96 (91.4)	126 (84.6)	0.104
Migraine	1 (1.6)	0 (0)	0.354	1 (1.0)	0 (0)	0.233
Obesity	28 (45.9)	21 (40.4)	0.555	41 (39.0)	44 (29.5)	0.113
Previous Stroke	20 (32.8)	22 (42.3)	0.297	35 (33.3)	43 (28.9)	0.447
Previous TIA (>24 h)	6 (9.8)	5 (9.6)	0.969	9 (8.6)	13 (8.7)	0.966
Prosthetic Heart Valve	1 (1.6)	2 (3.8)	0.467	1 (1.0)	4 (2.7)	0.328
Peripheral Vascular Disease	3 (4.9)	12 (23.1)	0.005 * ^a^	8 (7.6)	14 (9.4)	0.620
Chronic Renal Disease	10 (16.4)	6 (11.5)	0.461	22 (21.0)	23 (15.4)	0.257
Smoker	11 (18.0)	13 (25.0)	0.367	7 (6.7)	3 (2.0)	0.060
Medication History: No (%)						
HTN medication	50 (82.0)	46 (88.5)	0.336	98 (93.3)	135 (90.6)	0.437
Cholesterol Reducer	40 (65.6)	32 (61.5)	0.657	62 (59.0)	81 (54.4)	0.459
Diabetic Medication	25 (41.0)	23 (44.2)	0.728	33 (31.4)	37 (24.8)	0.247
Antidepressant	10 (16.4)	10 (19.2)	0.694	17 (16.2)	22 (14.8)	0.756
Lab values: Mean ± SD						
Total cholesterol	159.92 ± 43.21	164.49 ± 41.78	0.595	150.56 ± 45.16	156.35 ± 46.05	0.355
Triglycerides	149.98 ± 188.98	178.04 ± 189.47	0.463	119.70 ± 73.82	102 ± 46.12	0.050 * ^b^
HDL	41.60 ± 14.57	40.60 ± 14.88	0.736	41.37 ± 12.66	43.22 ± 13.11	0.299
LDL	94.52 ± 34.08	100.74 ± 35.21	0.374	86.84 ± 36.23	93.73 ± 38.42	0.180
Lipids	6.60 ± 1.69	6.66 ± 1.94	0.857	6.25 ± 1.35	6.20 ± 1.31	0.771
Blood Glucose	138.75 ± 64.03	153.73 ± 73.65	0.268	138.09 ± 57.06	143.19 ± 59.80	0.497
Serum Creatinine	1.52 ± 1.26	1.24 ± 0.61	0.154	1.35 ± 0.56	1.29 ± 0.59	0.477
INR	1.31 ± 0.53	1.19 ± 0.27	0.177	1.33 ± 0.58	1.24 ± 0.38	0.194
Vital Signs: Mean ± SD						
Heart Rate	83.3 ± 16.75	87.6 ± 22.67	0.267	76.9 ± 16.45	80.6 ± 16.97	0.084
Blood Pressure Systolic	142.21 ± 24.86	153.02 ± 30.87	0.042 * ^b^	147.98 ± 24.26	156.56 ± 28.59	0.010 * ^b^
Blood Pressure Diastolic	81.15 ± 16.48	83.90 ± 26.63	0.537	74.60 ± 15.15	79.92 ± 22.01	0.023 * ^b^
Ambulation Status Prior to Event: No. (%)						
Ambulate Independently	53 (86.9)	42 (80.8)	0.436	84 (80.0)	100 (67.1)	0.178
Ambulate with Assistance	3 (4.9)	1 (1.9)		10 (9.5)	17 (11.4)	
Unable to Ambulate	4 (6.6)	7 (13.3)		5 (4.8)	14 (9.4)	
Not Documented	1 (1.6)	2 (3.8)		6 (5.7)	17 (11.4)	
Ambulation Status on Admission: No. (%)						
Ambulate Independently	19 (31.1)	1 (1.9)	<0.001 * ^a^	18 (17.1)	0 (0)	<0.001 * ^a^
Ambulate with Assistance	26 (42.6)	7 (13.5)		49 (46.7)	15 (10.1)	
Unable to Ambulate	8 (13.1)	37 (71.2)		17 (16.2)	114 (76.5)	
Not Documented	8 (13.1)	7 (13.5)		21 (20.0)	20 (13.4)	
Ambulation Status on Discharge: No. (%)						
Ambulate Independently	32 (52.5)	7 (13.5)	<0.001 * ^a^	30 (28.6)	5 (3.4)	<0.001 * ^a^
Ambulate with Assistance	24 (39.3)	6 (11.5)		55 (52.4)	44 (29.5)	
Unable to Ambulate	4 (6.6)	30 (57.7)		17 (16.2)	75 (50.3)	
Not Documented	1 (1.6)	9 (17.3)		3 (2.9)	25 (16.8)	
rtPA Administration	9 (14.8)	19 (36.5)	0.008 * ^a^	15 (14.3)	41 (27.5)	0.012 * ^a^
Emergency Department	54 (88.5)	43 (82.7)	0.375	89 (84.8)	134 (89.9)	0.215
Direct Admission	7 (11.5)	9 (17.3)		16 (15.2)	15 (10.1)	
Improved Ambulation: No. (%)	19 (31.7)	12 (27.9)	0.682	33 (32.0)	46 (36.8)	0.452

Notes: ^a^ Pearson’s Chi-Squared test. ^b^ Student’s *T* test. * *p*-value < 0.05. BMI: Body Mass Index; HTN medication: Hypertensive medication; HDL: high-density lipoprotein; INR: international normalized ratio.

**Table 2:** Factors associated with stroke severity in the AIS population of Patients with Heart Failure between 65–74 and ≥75 years old. Adjusted OR < 1 denotes factors that are associated with AIS-HF patients in the 65–74 years old category, while OR > 1 denotes factors that are associated with AIS-HF patients ≥75 years old. Hosmer-Lemeshow test (*p* = 252), Cox & Snell *(R*^2^ = 0.112). The overall classified percentage of 63.0% was applied to check for the fitness of the logistic regression model. * Indicates statistical significance (*p* < 0.05) with a 95% confidence interval.

				95% C.I.		
Variables	B Value	Wald	Odds Ratio	Lower	Upper	*p*-Value
Drug and alcohol use	1.004	3.575	2.73	0.964	7.731	0.059
Peripheral Vascular Disease	1.124	8.404	3.079	1.439	6.585	0.004 *
Smoking	−0.867	8.787	0.420	0.237	0.745	0.003 *
Serum Creatinine	−0.398	7.134	0.672	0.502	0.900	0.008 *
Heart Rate	0.022	10.746	1.022	1.009	1.035	0.001 *
Systolic Blood Pressure	0.013	11.576	1.014	1.006	1.021	<0.001 *
Body Mass Index	−0.032	4.866	0.969	0.942	0.996	0.027 *
Direct Admission	−0.516	2.757	0.597	0.325	1.098	0.097

Notes: Backward Stepwise model based on Likelihood Ratio was applied. Model assumptions were fulfilled. Multicollinearity and interactions among independent variables were checked and no significant interactions were found. Hosmer-Lemeshow test (*p* = 0.252), Cox & Snell *(R*^2^ = 0.112).

**Table 3:** Factors associated with stroke severity in the AIS population of Patients with Heart Failure in the age category 65–74 years old. Adjusted OR < 1 denotes factors that are associated with a lower stroke severity (NIH Score ≤ 7), while OR > 1 denotes factors that are associated with a higher stroke severity (NIH Score > 7). Hosmer-Lemeshow test (*p* = 846), Cox & Snell *(R*^2^ = 0.229). The overall classified percentage of 70.2 % was applied to check for the fitness of the logistic regression model. * Indicates statistical significance (*p* < 0.05) with a 95% confidence interval.

				95% C.I.		
Variables	B Value	Wald	Odds Ratio	Lower	Upper	*p*-Value
Gender	−0.599	2.829	0.549	0.273	1.104	0.093
Coronary Artery Stenosis	1.202	2.753	3.328	0.804	13.767	0.097
Smoking	−2.257	6.257	0.105	0.018	0.614	0.012 *
Triglycerides	−0.007	5.548	0.993	0.987	0.999	0.019 *
INR	−0.790	3.408	0.454	0.196	1.050	0.045 *
Heart Rate	0.031	7.204	1.032	1.009	1.057	0.007 *
Direct admission	−1.035	4.542	0.355	0.137	0.920	0.033 *

Notes: Backward Stepwise model based on Likelihood Ratio was applied. Model assumptions were fulfilled. Multicollinearity and interactions among independent variables were checked and no significant interactions were found. Hosmer-Lemeshow test (*p* = 0.348), Cox & Snell *(R*^2^ = 0.141). INR: international normalized ratio.

**Table 4:** Factors associated with stroke severity in the AIS population of Patients with Heart Failure in the age category ≥75 years old. Adjusted OR<1 denotes factors that are associated with a lower stroke severity (NIH Score ≤ 7) while OR > 1 denotes factors that are associated with a higher stroke severity (NIH Score > 7). Hosmer-Lemeshow test (*p* = 0.348), Cox & Snell *(R*^2^ = 0.141). The overall classified percentage of 70.5 % was applied to check for the fitness of the logistic regression model. * Indicates statistical significance (*p* < 0.05) with a 95% confidence interval.

				95% C.I.		
Variables	B Value	Wald	Odds Ratio	Lower	Upper	*p*-Value
Previous Stroke	1.223	5.065	2.297	1.171	9.852	0.024 *
Peripheral Vascular Disease	1.915	2.547	6.784	1.242	37.065	0.027 *
Heart Rate	0.034	6.346	1.035	1.008	1.063	0.012 *
Systolic Blood Pressure	0.023	6.382	1.023	1.005	1.041	0.012 *

Notes: Backward Stepwise model based on Likelihood Ratio was applied. Model assumptions were fulfilled. Multicollinearity and interactions among independent variables were checked and no significant interactions were found. Hosmer-Lemeshow test (*p* = 0.846), Cox & Snell *(R*^2^ = 0.229).

## Data Availability

The retrospective datasets are available by request from the corresponding author of this manuscript, respectively.

## Abbreviations

Adjusted OR-: Adjusted odd ratio; atrial fibrillation: A fib, HRT: Hormone Therapy; BMI: Body mass index; CHF: Congestive heart failure; CI: Confidence interval; IRB: Institutional Review Board. INR: International normalized ratio; LDL-C: Low-density lipoprotein cholesterol; HDL-C: high-density lipoprotein cholesterol; rtPA: Recombinant tissue plasminogen; TC: Total cholesterol; TG: Triglyceride, AIS: Acute ischemic stroke; NIHSS: National Institute of Health Stroke Scale; MRI: Magnetic Resonance Imaging; CT: Computer Tomography; MCA: middle cerebral artery; CAD: coronary artery disease; HRT: hormone replacement therapy; TIA: transient ischemic attack; PVD: Peripheral vascular disease; ROC: Receiver Operating Curve; INR: International Normalized Ratio; HRV: heart rate variability; TP: total power; VLF; LF: low frequency; HF: high frequency domains.

## References

[1] Boehme A.K., Esenwa C., Elkind M.S. (2017). Stroke Risk Factors, Genetics, and Prevention. Circ. Res..

[2] Ovbiagele B., Nguyen-Huynh M.N. (2011). Stroke epidemiology: Advancing our understanding of disease mechanism and therapy. Neurotherapeutics.

[3] Roy-O’reilly M., Mccullough L.D. (2018). Age and Sex Are Critical Factors in Ischemic Stroke Pathology. Endocrinology.

[4] Avan A., Digaleh H., Di Napoli M., Stranges S., Behrouz R., Shojaeianbabaei G., Amiri A., Tabrizi R., Mokhber N., Spence J.D. (2019). Socioeconomic status and stroke incidence, prevalence, mortality, and worldwide burden: An ecological analysis from the Global Burden of Disease Study 2017. BMC Med..

[5] Reddy H.P. (2019). A study of age as a risk factor in ischemic stroke of elderly. Int. J. Med. Sci..

[6] Samuthpongtorn C., Jereerat T., Suwanwela N.C. (2021). Stroke risk factors, subtypes and outcome in elderly Thai patients. BMC Neurol..

[7] Yousufuddin M., Young N. (2019). Aging and ischemic stroke. Aging.

[8] Butrous H., Hummel S.L. (2016). Heart Failure in Older Adults. Can. J. Cardiol..

[9] Díez-Villanueva P., Jiménez-Méndez C., Alfonso F. (2021). Heart failure in the elderly. J. Geriatr. Cardiol. JGC.

[10] Fleming T., Blum B., Averkamp B., Sullivan J., Nathaniel T. (2019). Effect of antihypertensive medications on thrombolysis therapy and outcomes in acute ischemic stroke patients. J. Clin. Hypertens..

[11] Gainey J., Blum B., Bowie B., Cooley K., Madeline L., Ervin E.L., Nathaniel T.I. (2018). Stroke and dyslipidemia: Clinical risk factors in the telestroke versus non-telestroke. Lipids Health Dis..

[12] Chen S., Che Q., Zheng Q., Zhang Y., Jia J., Wu Y., Huo Y., Chen D. (2021). Relationship Between Different Risk Factor Patterns and Follow-Up Outcomes in Patients with ST-Segment Elevation Myocardial Infarction. Front. Cardiovasc. Med..

[13] Pullicino P.M., McClure L.A., Wadley V.G., Ahmed A., Howard V.J., Howard G., Safford M.M. (2009). Blood pressure and stroke in heart failure in the REasons for Geographic And Racial Differences in Stroke (REGARDS) study. Stroke.

[14] Kim J.-S., Lee K.-B., Roh H., Ahn M.-Y., Hwang H.-W. (2010). Gender differences in the functional recovery after acute stroke. J. Clin. Neurol..

[15] Degraba T.J., Hallenbeck J.M., Pettigrew K.D., Dutka A.J., Kelly B.J. (1999). Progression in acute stroke*—*Value of the initial NIH Stroke Scale score on patient stratification in future trials. Stroke.

[16] Rangaraju S., Jovin T.G., Frankel M., Schonewille W.J., Algra A., Kappelle L.J., Nogueira R.G., BASICS Study Group (2016). Neurologic Examination at 24 to 48 Hours Predicts Functional Outcomes in Basilar Artery Occlusion Stroke. Stroke.

[17] Edrissi C., Rathfoot C., Knisely K., Sanders C.B., Poupore N., Nathaniel T. (2021). Gender disparity in a cohort of stroke patients with incidence of obstructive sleep apnea. J. Vasc. Nurs..

[18] Howard G., Goff D.C. (2012). Population shifts and the future of stroke: Forecasts of the future burden of stroke. Ann. N. Y. Acad. Sci..

[19] Nakanishi K., Di Tullio M.R., Qian M., Thompson J.L., Labovitz A.J., Mann D.L., Sacco R.L., Pullicino P.M., Freudenberger R.S., Teerlink J.R. (2017). Resting Heart Rate and Ischemic Stroke in Patients with Heart Failure. Cerebrovasc. Dis..

[20] Rathfoot C., Edrissi C., Sanders C.B., Knisely K., Poupore N., Nathaniel T. (2021). Gender differences in comorbidities and risk factors in ischemic stroke patients with a history of atrial fibrillation. BMC Neurol..

[21] Yamamoto M., Ohta Y., Sakuma M., Takeuchi J., Matsumoto C., Morimoto T. (2019). Association between heart rate on admission and in-hospital mortality among general inpatients: Insights from Japan Adverse Drug Events (JADE) study. Medicine.

[22] Kotecha D., Flather M.D., Altman D.G., Holmes J., Rosano G., Wikstrand J., Packer M., Coats A.J.S., Manzano L., Böhm M. (2017). Heart Rate and Rhythm and the Benefit of Beta-Blockers in Patients with Heart Failure. J. Am. Coll. Cardiol..

[23] Nikolovska Vukadinović A., Vukadinović D., Borer J., Cowie M., Komajda M., Lainscak M., Swedberg K., Böhm M. (2017). Heart rate and its reduction in chronic heart failure and beyond. Eur. J. Heart Fail..

[24] Lee K.J., Kim B.J., Han M.K., Kim J.-T., Choi K.-H., Shin D.-I., Yeo M.-J., Cha J.-K., Kim D.-H., Nah H.-W. (2020). Effect of Heart Rate on Stroke Recurrence and Mortality in Acute Ischemic Stroke with Atrial Fibrillation. Stroke.

[25] Chen J., Li S., Zheng K., Wang H., Xie Y., Xu P., Dai Z., Gu M., Xia Y., Zhao M. (2019). Impact of Smoking Status on Stroke Recurrence. J. Am. Heart Assoc..

[26] Lee J.-D., Kuo Y.-W., Lee C.-P., Huang Y.-C., Lee M., Lee T.-H. (2022). Initial in-hospital heart rate is associated with long-term survival in patients with acute ischemic stroke. Clin. Res. Cardiol..

[27] Poupore N., Strat D., Mackey T., Snell A., Nathaniel T. (2019). Ischemic stroke with a preceding Trans ischemic attack(TIA) less than 24 hours: Predicting clinical risk factors for thrombolytic therapy. BMC Neurol..

[28] Okon M., Blum B., Nathaniel T.I. (2021). Risk factors and ambulatory outcome in ischemic stroke patients with pre-stroke depression. J. Vasc. Nurs. Off. Publ. Soc. Peripher. Vasc. Nurs..

[29] Can A., Castro V.M., Dligach D., Finan S., Yu S., Gainer V., Shadick N.A., Savova G., Murphy S., Cai T. (2018). Elevated International Normalized Ratio Is Associated with Ruptured Aneurysms. Stroke.

[30] Jauch E.C., Saver J.L., Adams H.P., Bruno A., Connors J.J., Demaerschalk B.M., Khatri P., McMullan P.W., Qureshi A.I., Rosenfield K. (2013). Guidelines for the early management of patients with acute ischemic stroke: A guideline for healthcare professionals from the American Heart Association/American Stroke Association. Stroke.

[31] Awujoola A., Sodeke P., Olufeyisayo O., Mokikan M., Adeyemi E., Babalola G., Awujoola O., Okon M., Nathaniel T.I. (2021). Clinical Risk Factors Associated with Ambulatory Outcome in Acute Ischemic Stroke Patients Smokers Treated with Thrombolytic Therapy. Am. J. Med. Sci..

[32] Gainey J., Brecthtel L., Blum B., Keels A., Madeline L., Lowther E., Nathaniel T. (2018). Functional Outcome Measures of Recombinant Tissue Plasminogen Activator-Treated Stroke Patients in the Telestroke Technology. J. Exp. Neurosci..

[33] Okon M., Adebobola N.I., Julius S., Adebimpe O., Taiwo A.O., Akinyemi A., Thomas N.I. (2015). Stroke Incidence and Case Fatality Rate in an Urban Population. J. Stroke Cerebrovasc. Dis..

[34] Seiffge D.J., De Marchis G.M., Koga M., Paciaroni M., Wilson D., Cappellari M., Macha K., Tsivgoulis G., Ambler G., Arihiro S. (2020). Ischemic Stroke despite Oral Anticoagulant Therapy in Patients with Atrial Fibrillation. Ann. Neurol..

[35] Cao C., Martinelli A., Spoelhof B., Llinas R.H., Marsh E.B. (2017). In Potential Stroke Patients on Warfarin, the International Normalized Ratio Predicts Ischemia. Cerebrovasc. Dis. Extra.

[36] Dager W.E., Branch J.M., King J.H., White R.H., Quan R.S., Musallam N.A., Albertson T.E. (2000). Optimization of inpatient warfarin therapy: Impact of daily consultation by a pharmacist-managed anticoagulation service. Ann. Pharmacother..

[37] Zhao Z., Zhao Z., Zheng X., Li X., Li X., Huang C., Shan Y., Nyame L., Ibrahim M., Gao X. (2020). The association between smoking and unfavorable outcomes in acute ischemic stroke patients with mechanical thrombectomy. Tob. Induc. Dis..

[38] Rotimi O.R., Ajani I.F., Penwell A., Lari S., Walker B., Nathaniel T.I. (2020). In acute ischemic stroke patients with smoking incidence, are more women than men more likely to be included or excluded from thrombolysis therapy?. Women’s Health.

[39] Li B., Li D., Liu J.F., Wang L., Li B.-Z., Yan X.-J., Liu W., Wu K., Xiang R.-L. (2021). “Smoking paradox” is not true in patients with ischemic stroke: A systematic review and meta-analysis. J. Neurol..

[40] Luo J., Tang X., Li F., Wen H., Wang L., Ge S., Tang C., Xu N., Lu L. (2021). Cigarette Smoking and Risk of Different Pathologic Types of Stroke: A Systematic Review and Dose-Response Meta-Analysis. Front. Neurol..

[41] Kurmann R., Engelter S.T., Michel P., Luft A.R., Wegener S., Branscheidt M., Eskioglou E., Sirimarco G., Lyrer P.A., Gensicke H. (2018). Impact of Smoking on Clinical Outcome and Recanalization After Intravenous Thrombolysis for Stroke: Multicenter Cohort Study. Stroke.

[42] Shah R.S., Cole J.W. (2010). Smoking and stroke: The more you smoke the more you stroke. Expert Rev. Cardiovasc. Ther..

[43] Song Y., Kim J.-G., Cho H.-J., Kim J.K., Suh D.C. (2017). Evaluation of cerebral blood flow change after cigarette smoking using quantitative MRA. PLoS ONE.

[44] Thomas S., Makris M. (2018). The reversal of anticoagulation in clinical practice. Clin. Med..

[45] Nathaniel T., Otukonyong E., Okon M., Chaves J., Cochran T., Imeh-Nathaniel A. (2013). Metabolic regulatory clues from the naked mole rat: Toward brain regulatory functions during stroke. Brain Res. Bull..

[46] Kufner A., Nolte C.H., Galinovic I., Brunecker P., Kufner G.M., Endres M., Fiebach J.B., Ebinger M. (2013). Smoking-Thrombolysis Paradox Recanalization and Reperfusion Rates After Intravenous Tissue Plasminogen Activator in Smokers with Ischemic Stroke. Stroke.

[47] Simundic A.-M., Nikolac N., Basic-Kes V., Demarin V.J.C.C., Medicine L. (2008). Are serum lipids measured on stroke admission prognostic?. Clin. Chem. Lab. Med..

[48] Li W., Liu M., Wu B., Liu H., Wang L.-C., Tan S. (2008). Serum lipid levels and 3-month prognosis in Chinese patients with acute stroke. Adv. Ther..

[49] Greene S.J., Vaduganathan M., Lupi L., Ambrosy A.P., Mentz R.J., Konstam M.A., Nodari S., Subacius H.P., Fonarow G.C., Bonow R.O. (2013). Prognostic significance of serum total cholesterol and triglyceride levels in patients hospitalized for heart failure with reduced ejection fraction (from the EVEREST Trial). Am. J. Cardiol..

[50] Brechtel L., Poupore N., Monroe M., Knisely K., Sanders C., Edrissi C., Rathfoot C., Nathaniel T.I. (2021). Role of dyslipidemia in ischemic stroke patients treated in the telestroke network. Adv. Med. Sci..

[51] Kozdag G., Ertas G., Emre E., Akay Y., Celikyurt U., Sahin T., Gorur G., Karauzum K., Yilmaz I., Ural D. (2013). Low serum triglyceride levels as predictors of cardiac death in heart failure patients. Tex. Heart Inst. J..

[52] Brechtel L., Poupore N., Stoikov T., Roley L.T., Emerson J., Nathaniel I.T. (2020). Comorbidities associated with different levels of total cholesterol in male and female acute ischemic stroke patients. Medicine.

[53] Anni N.S., Jung S.J., Shim J.-S., Jeon Y.W., Lee G.B., Kim H.C. (2021). Stressful life events and serum triglyceride levels: The Cardiovascular and Metabolic Diseases Etiology Research Center cohort in Korea. Epidemiol. Health.

[54] Dong J., Yang S., Zhuang Q., Sun J., Wei P., Zhao X., Chen Y., Chen X., Li M., Wei L. (2021). The Associations of Lipid Profiles with Cardiovascular Diseases and Death in a 10-Year Prospective Cohort Study. Front. Cardiovasc. Med..

[55] Freiberg J.J., Tybjærg-Hansen A., Jensen J.S., Nordestgaard B.G. (2008). Nonfasting Triglycerides and Risk of Ischemic Stroke in the General Population. JAMA.

[56] Siedler G., Sommer K., Macha K., Marsch A., Breuer L., Stoll S., Engelhorn T., Dörfler A., Arnold M., Schwab S. (2019). Heart Failure in Ischemic Stroke: Relevance for Acute Care and Outcome. Stroke.

[57] Bekelis K., Marth N.J., Wong K., Zhou W., Birkmeyer J.D., Skinner J. (2016). Primary Stroke Center Hospitalization for Elderly Patients with Stroke: Implications for Case Fatality and Travel Times. JAMA Int. Med..

[58] Kim D.H., Bae H.J., Han M.K., Kim B.J., Park S.-S., Park T.H., Lee K.B., Kang K., Park J.-M., Ko Y. (2016). Direct admission to stroke centers reduces treatment delay and improves clinical outcome after intravenous thrombolysis. J. Clin. Neurosci. Off. J. Neurosurg. Soc. Australas..

[59] Pérez De La Ossa N., Millán M., Arenillas J.F., Sánchez-Ojanguren J., Palomeras E., Dorado L., Guerrero C., Dávalos A. (2009). Influence of direct admission to Comprehensive Stroke Centers on the outcome of acute stroke patients treated with intravenous thrombolysis. J. Neurol..

[60] Qin H., Wang P., Zhang R., Yu M., Zhang G., Liu G., Wang Y. (2020). Stroke History is an Independent Risk Factor for Poor Prognosis in Ischemic Stroke Patients: Results from a Large Nationwide Stroke Registry. Curr. Neurovasc. Res..

[61] Nathaniel T., Sanders C.B., Knisely K., Edrissi C., Rathfoot C., Poupore N., Wormack L. (2021). Obstructive sleep apnea and stroke severity: Impact of clinical risk factors. Brain Circ..

[62] Poupore N., Strat D., Mackey T., Brown K., Snell A., Nathaniel T.I. (2020). Cholesterol reducer and thrombolytic therapy in acute ischemic stroke patients. Lipids Health Dis..

[63] Polk S., Stafford C., Adkins A., Efird J., Colello M., Nathaniel I.T. (2018). Contraindications with recombinant tissue plasminogen activator (rt-PA) in acute ischemic stroke population. Neurol. Psychiatry Brain Res..

[64] Poupore N., Strat D., Mackey T., Nathaniel I.T. (2020). The Association Between an Antecedent of Transient Ischemic Attack Prior to Onset of Stroke and Functional Ambulatory Outcome. Clin. Appl. Thromb./Hemost..

[65] Caro J., Migliaccio-Walle K., Ishak K.J., Proskorovsky I. (2005). The morbidity and mortality following a diagnosis of peripheral arterial disease: Long-term follow-up of a large database. BMC Cardiovasc. Disord..

[66] Meves S.H., Diehm C., Berger K., Pittrow D., Trampisch H.-J., Burghaus I., Tepohl G., Allenberg J.-R., Endres H.G., Schwertfeger M. (2010). Peripheral arterial disease as an independent predictor for excess stroke morbidity and mortality in primary-care patients: 5-year results of the getABI study. Cerebrovasc. Dis..

[67] Pirson F.a.V., Hinsenveld W.H., Staals J., De Ridder I.R., Van Zwam W.H., Schreuder T.H.C.M.L., Roos Y.B.W.E.M., Majoie C.B.L.M., Van Der Worp H.B., Uyttenboogaart M. (2020). Peripheral Artery Disease in Acute Ischemic Stroke Patients Treated with Endovascular Thrombectomy; Results From the MR CLEAN Registry. Front. Neurol..

[68] Whelton P.K., Carey R.M., Aronow W.S., Casey D.E., Collins K.J., Himmelfarb C.D., DePalma S.M., Gidding S., Jamerson K.A., Jones D.W. (2018). 2017 ACC/AHA/AAPA/ABC/ACPM/AGS/APhA/ASH/ASPC/NMA/PCNA Guideline for the Prevention, Detection, Evaluation, and Management of High Blood Pressure in Adults. J. Am. Coll. Cardiol..

